# Periodic Fever and Neutrophilic Dermatosis: Is It Sweet's Syndrome?

**DOI:** 10.1155/2014/320920

**Published:** 2014-12-04

**Authors:** Raheleh Assari, Vahid Ziaee, Nima Parvaneh, Mohammad-Hassan Moradinejad

**Affiliations:** ^1^Division of Pediatric Rheumatology, Children's Medical Center, Pediatrics Center of Excellence, Tehran 14194, Iran; ^2^Pediatric Rheumatology Research Group, Rheumatology Research Center, Tehran University of Medical Sciences, Tehran, Iran; ^3^Department of Pediatrics, Tehran University of Medical Sciences, Tehran, Iran; ^4^Research Center for Immunodeficiencies, Tehran University of Medical Sciences, Tehran, Iran

## Abstract

A 7-year-old boy with high grade fever (39°C) and warm, erythematous, and indurated plaque above the left knee was referred. According to the previous records of this patient, these indurated plaques had been changed toward abscesses formation and then spontaneous drainage had occurred after about 6 to 7 days, and finally these lesions healed with scars. In multiple previous admissions, high grade fever, leukocytosis, and a noticeable increase in erythrocyte sedimentation rate and C-reactive protein were noted. After that, until 7th year of age, he had shoulder, gluteal, splenic, kidney, and left thigh lesions and pneumonia. The methylprednisolone pulse (30 mg/kg) was initiated with the diagnosis of Sweet's syndrome. After about 10–14 days, almost all of the laboratory data regressed to nearly normal limits. After about 5 months, he was admitted again with tachypnea and high grade fever and leukocytosis. After infusion of one methylprednisolone pulse, the fever and tachypnea resolved rapidly in about 24 hours. In this admission, colchicine (1 mg/kg) was added to the oral prednisolone after discharge. In the periodic fever and neutrophilic dermatosis, the rheumatologist should search for sterile abscesses in other organs.

## 1. Introduction

Neutrophilic dermatosis (ND) is a group of disorders with intense neutrophilic infiltration in the skin and extracutaneous involvement. Recently, these disorders have been known as neutrophilic diseases [1]. ND is presented as dermal neutrophilic dermatosis (such as Sweet's syndrome), dermal and hypodermal neutrophilic dermatosis (pyoderma gangrenosum, neutrophilic panniculitis, and skin aseptic abscesses), and epidermal neutrophilic dermatosis [1]. ND may be associated with other systemic disorders such as myeloproliferative disorders, inflammatory bowel disease, and rheumatoid arthritis [1]. In the children, ND may precede the other manifestations of underlying disease for many years [2]. Familial Mediterranean fever and ND have similar clinical manifestations that suggest the possibility of similar mechanism to stimulate neutrophils [3].

In this paper, a case of periodic fever associated with ND (dermal, dermal and hypodermal neutrophilic dermatoses) and hyperleukocytosis, initiated from the neonatal period, was reported.

## 2. Case Report

**Figure 1:**
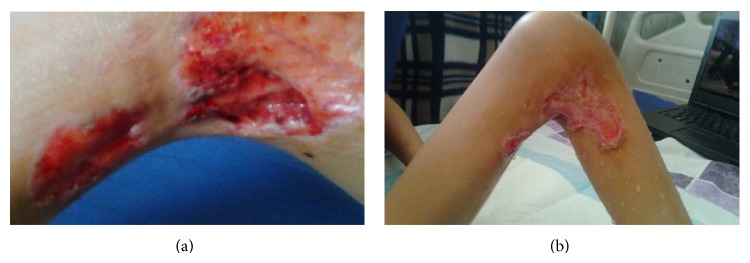
Skin lesion in the left knee in our patients with Sweet's syndrome: (a) acute phase, (b) after 7 days.

A 7-year-old boy was referred to the rheumatology clinic with high grade fever (*T* = 39°C) and warm, erythematous, and indurated plaque above the left knee with the size of about 8 × 10 cm diameters (Figure 1). According to the previous records of this patient, these indurated plaques had been changed toward abscesses formation and then spontaneous drainage had occurred after about 6 to 7 days, and finally these lesions healed with scars. He had two scars of previous lesions on the outer side of the left thigh and the outer side of right shoulder.

He was born at 33rd week of gestational age, with about 2130 g birth weight. He had low Apgar scores at the first and fifth minutes of the birth time. He received surfactant due to prematurity of the lungs. He stayed in hospital for about one month. During the admission he had fever and plantar cellulitis, cellulitis of the right testis, and abscess and necrosis of the root of umbilical cord. So, he received a combination of meropenem and vancomycin antibiotics. The smears and cultures of these lesions were negative. The histopathology of plantar cellulitis represented granulation tissues with nonspecific inflammation. The umbilical cord had delay in seperation for about 45 days. So, the clinicians decided to cut it off. The first complete blood count of his life revealed white blood cell count (WBC) of 9700 per mm^3^, with 26% neutrophils and 61% lymphocytes, hemoglobin (Hb) of 13.4 g/dL, and the platelet (PLT) count of 443000 per mm^3^. During the first year of life, he had seven admissions for recurrent respiratory infections.

After the first year, he had repetitive admissions due to fever and leukocytosis. He had history of splenic lesions with left lobar pneumonia in second year. About 2 months later, a lung lesion appeared in the left upper lobe with about 10 × 10 cm size. After that, until 7th year of age, he had shoulder lesion, gluteal lesion, splenic lesions, kidney lesions (enlargement of both kidneys with multiple low-density areas in ultrasonography), left thigh lesion, and pneumonia. In one of these admissions, he presented with pyoderma gangrenosum (PG) and chest wall lesion. In another admission, the erythema nodosum-like lesions on the feet appeared. These recurrences were usually preceded by upper respiratory tract or gastrointestinal infections.

The laboratory data in one admission with abscesses-like formation in upper lobe of right lung revealed white blood cell count 68300 per mm^3^, with 82% neutrophils and 10% lymphocytes, hemoglobin 7 g/dL, platelet count 694000 per mm^3^, ESR 103 mm/hour, and CRP 78 mg/dL. After treatment with broad spectrum antibiotics for about 10–14 days, the laboratory data demonstrated WBC 10100 per mm^3^, with 25% neutrophils, and 65% lymphocytes, Hb 8.9 g/dL, PLT 724000 per mm^3^, ESR 41 mm/hour, and CRP 6.1 mg/dL. In all admissions, high grade fever, leukocytosis, and a noticeable increase in erythrocyte sedimentation rate and C-reactive protein were noted. After about 10–14 days, almost all of the laboratory data regressed to nearly normal limits.

In these seven years, all of the cultures and smears for infections were negative. Smears of the lesions showed many white blood cells with 95% neutrophils and 5% lymphocytes. In evaluation for immunodeficiency disorders, CD3, CD4, CD8, CD16, CD19, and CD56 were within the normal limits. The nitroblue tetrazolium (NBT) test was 100 percent. The genetic evaluations for the leukocyte adhesion deficiency (LAD) were negative. In the bone marrow aspiration, the proportion of the myeloid to erythroid increased to 30–40/1, with no evidence of malignancy. The autoantibodies [antinuclear antibodies, rheumatoid factor, and antimyeloperoxidase-antineutrophil cytoplasmic antibody (ANCA)] were negative. The serum levels of immunoglobulins IgG, IgM, IgA, C3, C4, CH50, and angiotensin-converting enzyme (ACE) were normal.

In spite of these results, at the end of 3rd year of life, with suspicion of the immunodeficiency disorders, intravenous immune globulin (IVIG) was initiated monthly. Also, trimethoprim-sulfamethoxazole was added for the prophylaxis. With this treatment, the intervals between the recurrences were prolonged.

The methylprednisolone pulse (30 mg/kg) was initiated with the diagnosis of Sweet's syndrome (SS). Then, all of the signs and symptoms improved rapidly: the fever resolved in less than 24 hours, the lesions did not progress to abscess and resolved without any scars. After discharge, prednisolone with the dose of 0.5–0.75 mg/kg/day was prescribed.

**Figure 2:**
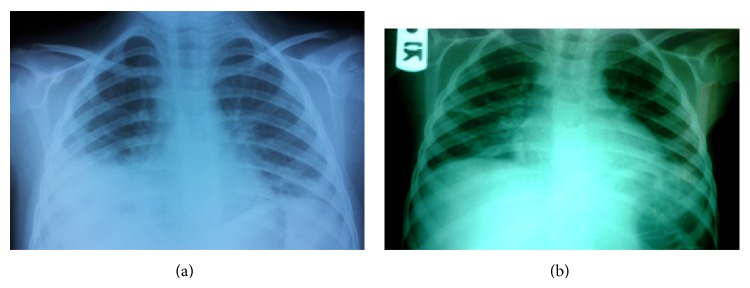
Chest X-ray in our patients with Sweet's syndrome: (a) pleural effusion in acute phase before treatment, (b) after methylprednisolone pulse therapy.

After about 5 months, he was admitted again with tachypnea and high grade fever and leukocytosis. In this admission, he did not have any skin lesion. Chest X-ray showed pleural effusion in both sides especially in the right side (Figure 2). After infusion of one methylprednisolone pulse, the fever and tachypnea resolved rapidly in about 24 hours. The leukocytosis improved in less than 7 days. In this admission, the colchicine (1 mg/kg) was added to the oral prednisolone after discharge. The prednisolone was tapered slowly (0.25 mg/kg/d every 2 months). Fortunately, our patient had no flare-up after 6 months.

## 3. Discussion

Neutrophilic dermatosis (ND) is a sterile inflammation of skin with normal polymorphonuclear leukocytes [1]. Other organs such as lung, heart, blood vessels, liver, spleen, pancreas, and central nervous system may be affected by the sterile infiltration [4]. Furthermore, this infiltration can present as sterile abscess in other organs [5]. So, neutrophilic dermatosis should be proposed as a neutrophilic systemic disease [1]. Histological lesions, the associated peripheral neutrophilia, and the possible reaction to drugs that disturb the neutrophils function supported the potential role of neutrophils in these disorders [6]. Exogenous granulocyte colony stimulating factor (G-CSF) that is used in the treatment of low-neutrophil count disorders suppresses apoptosis and increases survival of neutrophils. Thus, despite low absolute neutrophil counts, the accumulation of neutrophils in skin lesion increased rapidly and the ND manifestations may develop [7]. Increased tyrosine phosphorylation of the proteins in the neutrophils of ND suggests that the possible defect in the tyrosine phosphatase function can prolong the survival of neutrophils [8, 9]. Our patient had high levels of leukocytosis during attacks. So, the numbers of neutrophils might also play a role in the manifestations.

Nowadays, the ND has been proposed as an autoinflammatory disorder [10, 11]. In the autoinflammatory diseases, a number of genetic defects in the innate immune system [12] cause recurrent episodes of sterile inflammation in the skin and other organs without high titers of autoantibodies or autoreactive T cells [13]. Alternatively, overproduction of the IL-1*β* can release cytokines and chemokines, causing neutrophilic stimulation [14].

On the other hand, IL-1 antagonist has been used in the treatment of autoinflammatory diseases (such as cryopyrin-related periodic disease). Moreover, other than corticosteroids (the main part of treatment in ND), IL-1 antagonist (anakinra) has recently been used in the treatment of SS [15]. So, the response to the IL-1 antagonist could be a diagnostic criterion for autoinflammatory diseases [1].

Autoinflammatory diseases were considered monogenic, such as PAPA (pyogenic sterile arthritis, PG, and acne) syndrome in which a mutation in the* PSTPIP1* gene causes overproduction of IL1 and resultant neutrophil-mediated reactions in skin and organs [16]. Neutrophilic diseases share some clinical features with monogenic autoinflammatory disorders such as periodic fever and neutrophil infiltration in the skin and other organs. The SS was reported in monogenic disorders, such as Majeed syndrome [17]. But the differences in clinical manifestations, even within one group of ND, diversity in the subtypes of ND, and differences in the response to therapy [8] support the concept that neutrophilic diseases may be a new category named polygenic autoinflammatory diseases [1].

According to the von den Driesch diagnostic criteria in Sweet's syndrome [18], our patient had all minor criteria (fever about 39°C, history of upper respiratory tract infections in some recurrences, gastrointestinal infection in one episode, good response to corticosteroid after 36–48 hours, high levels of white blood cells, neutrophils, ESR, and C-reactive protein). The characteristic feature of our patient in laboratory data was high levels of leukocytosis (above 50,000 per mm^3^) in each episode. When the WBC count exceeds 50000 per mm^3^, the term of leukemoid reaction is used (due to the similarity to leukemia). Most frequently, this term was associated with septicemia and severe bacterial infections such as shigellosis, salmonellosis, and meningococcemia [19]. There was only one report of leukemoid reaction and PG in two middle-aged women [20]. As we know, our report is the first description of the ND and periodic fever associated with leukemoid reaction. Some factors other than cytokines may stimulate neutrophil productions in this disorder and cause leukemoid reaction.

According to the von den Driesch major criteria, our patient had dense neutrophilic infiltration without any vasculitis in the histopathology and tender erythematous plaques or atypical bullous lesions. Furthermore, in one episode, our patient had pyoderma gangrenosum on the lower extremities. In the malignancy-associated SS, the lesions similar to PG were reported [21]. In the other episode, erythema nodosum-like lesions were observed. The subcutaneous neutrophil infiltration with tender dermal nodules similar to EN was also reported in SS [22]. Some episodes were concurrent with skin aseptic abscesses that were not characteristic of SS.

The SS in children is often classified in the classic category, which is idiopathic and triggered by the upper respiratory tract or gastrointestinal infections [23]. In the neonatal SS, the workup for immunodeficiency diseases (neutrophil dysfunction and antibody testing), viral infections (especially HIV), and hematologic diseases should be performed [2]. In addition, the evidences of monogenic autoinflammatory disorders such as CANDLE syndrome (chronic atypical neutrophilic dermatosis, lipodystrophy, and elevated temperature) and familial Sweet's syndrome should be sought in a case with neonatal SS [2].

Aseptic abscesses were reported in some cases with neutrophilic dermatosis or inflammatory bowel disease. The aseptic abscess (AA) syndrome was also described, when organ involvements with sterile abscesses were prominent [23]. These two categories may represent one autoinflammatory disease with different features. Most cases of AA syndrome were young adults and had a rapid response to corticosteroid therapy [24]. Recently, TNF-*α* blockade [25, 26] has been used in the treatment of AA syndrome. The effectiveness of these treatments is still unclear and should be evaluated with more studies.

Although corticosteroids have rapid responses in ND, the recurrences occur in the course of tapering [2]. Colchicine was proposed for the treatment of ND or SS with no relapses in follow-up [26, 27] (like our patient).

To our knowledge, our report was the first report of ND with multiple skin presentations from the neonatal period. Evaluation of all reported cases and further genetic investigations may lead to the diagnosis of a new autoinflammatory syndrome.

In conclusion, the rheumatologists should be aware of this condition when encountering periodic fever and neutrophilic dermatosis and should search for sterile abscesses in other organs. Moreover, in neonatal leukemoid reaction, when infections, malignancies, and immunodeficiencies are ruled out, the neutrophilic dermatosis and other autoinflammatory diseases should be considered.
